# Body Composition Metrics Associated with Time to Progression in Smoldering Multiple Myeloma

**DOI:** 10.3390/diagnostics15212760

**Published:** 2025-10-31

**Authors:** Fabian Bauer, Florian A. Huber, Marilyn E. Galdamez, Ivanna Zorgno, Sina Habibollahi, Amine El Kandoussi, Florian J. Fintelmann, P. Erik Tonnesen, Anna-Sophia W. Dietrich, Zhe Wang, Adam Graeber, Robert D. Boutin, Leon Lenchik, Joshua N. Gustine, Steven J. Staffa, Noopur Raje, Connie Y. Chang

**Affiliations:** 1 Division of Musculoskeletal Imaging and Intervention, Department of Radiology, Massachusetts General Hospital and Harvard Medical School, Boston, MA 02114, USAcychang@mgh.harvard.edu (C.Y.C.); 2 Division of Radiology, German Cancer Research Center, 69120 Heidelberg, Germany; 3 Institute for Diagnostic and Interventional Radiology, Faculty of Medicine and University Hospital Cologne, University of Cologne, 50937 Cologne, Germany; 4 Institute of Diagnostic and Interventional Radiology, University Hospital Zurich, Faculty of Medicine, University of Zurich, 8091 Zurich, Switzerland; 5 Metabolic Imaging Core, Nutrition Obesity Research Center at Harvard, Massachusetts General Hospital, Harvard Medical School, Boston, MA 02114, USA; 6 Cardiovascular Imaging Research Center, Department of Radiology, Massachusetts General Hospital, Harvard Medical School, Boston, MA 02114, USA; 7 Department of Radiology, Yale New Haven Health- Bridgeport Hospital, Bridgeport, CT 06610, USA; 8 Division of Thoracic Imaging and Intervention, Department of Radiology, Massachusetts General Hospital and Harvard Medical School, Boston, MA 02114, USA; 9 Department of Radiology and Nuclear Medicine, University Hospital Schleswig-Holstein, 23538 Lubeck, Germany; 10 Department of Internal Medicine I, University Hospital Frankfurt, Goethe University, 60590 Frankfurt am Main, Germany; 11 Department of Radiology, Brooke Army Medical Center, Fort Sam Houston, TX 78234, USA; adamgraeber@gmail.com; 12 Department of Radiology, University of California Davis School of Medicine, Sacramento, CA 95817, USA; 13 Department of Radiology, Wake Forest University School of Medicine, Medical Center Boulevard, Winston-Salem, NC 27157, USA; 14 Center for Multiple Myeloma, Massachusetts General Hospital Cancer Center and Harvard Medical School, Boston, MA 02114, USAnraje@mgh.harvard.edu (N.R.); 15 Department of Anesthesiology and Surgery, Boston Children’s Hospital, Boston, MA 02115, USA

**Keywords:** smoldering multiple myeloma, body composition, computed tomography, time to progression

## Abstract

**Objective**: To determine the association of body composition (BC) in smoldering multiple myeloma (SMM) with time to progression (TTP) to MM. **Methods**: The quantity and quality of adipose and muscle tissue were retrospectively derived from 63 whole-body low-dose computed tomography (WBLDCT) scans between 2017 and 2021. BC was analyzed by segmenting a single axial image at the level of the fourth lumbar vertebrae. Subjects were grouped into below vs. above the sex-specific median for BC metrics. Clinical information including TTP and progression risk factors were recorded. Cox proportional hazard models were used to determine the association between BC metrics and TTP. BC groups were compared using the Wilcoxon rank sum test and Fisher’s exact test. **Results**: Thirty subjects progressed over a median follow-up of 49.2 months. For subjects with a subcutaneous adipose tissue (SAT) cross-sectional area (CSA) below vs. above the median, TTP was 24.8 vs. not reached (*p* = 0.02). Similarly, TTP was 20.7 vs. not reached (*p* = 0.01) for those with SAT CSA indexed to height below vs. above the median. High SAT CSA (hazard ratio [HR]: 0.42 [95%CI: 0.20–0.90], *p* = 0.03) and high SAT index (HR: 0.39 [95%CI: 0.18–0.83], *p* = 0.01) were both associated with a lower progression risk. High SAT index remained significantly associated with reduced progression risk in multivariate analysis (*p* = 0.03). There was no association between TTP and obesity (BMI ≥ 30 kg/m^2^) or muscle metrics. High SAT CSA and index were associated with younger age and higher hemoglobin levels. **Conclusions**: SAT quantity might serve as a prognostic marker for progression in SMM.

## 1. Introduction

Smoldering multiple myeloma (SMM) is a precursor to multiple myeloma (MM) with varying time to progression (TTP) and outcomes [1]. In SMM, median TTP is 5 years with 73% of SMM patients progressing within 15 years of diagnosis [2]. Current management strategies of precursor diseases rely on evaluating patient characteristics and myeloma-specific factors predictive of progression to MM [3,4,5,6,7,8,9]. The Mayo 20/2/20 risk stratification system categorizes SMM patients into distinct risk groups based on a serum-free light chain ratio (SFLCR) of more than 20, a monoclonal protein (m-protein) concentration of more than 2.0 g/dL, and plasma cell infiltration of more than 20% [7]. An updated version includes high-risk cytogenetic aberrations as an additional criterion [8]. Recently, the PANGEA models demonstrated superior predictive accuracy for progression in monoclonal gammopathy of unknown significance (MGUS) and SMM by incorporating time-varying biomarkers such as SFLCR, m-protein, serum creatinine, hemoglobin, age, and optionally plasma cell infiltration [3].

To rule out myeloma-defining lytic lesions [10], the International Myeloma Working Group (IMWG) recommends whole-body low-dose CT (WBLDCT) as the first-line imaging modality for suspected (S)MM and high-risk MGUS [11]. Beyond providing the diagnosis of myeloma, WBLDCT also offers opportunistic insights into body composition (BC). BC is typically assessed using a single cross-sectional CT image at the L3 or L4 lumbar level to estimate skeletal muscle and adipose tissue quality and quantity [12]. Sarcopenia, characterized by low muscle mass, function, or quality, has been previously established as a significant predictor of morbidity and mortality across various cancer entities [13,14,15,16,17,18]. In MM, various studies have evaluated the association between sarcopenia and disease features or outcomes, reporting discordant results [19,20,21,22,23,24,25,26,27]. Similarly, BC metrics capturing the distribution and quality of abdominal adipose tissue were investigated for their impact on outcome, again with diverging results [19,20,24,26,28]. While BC analysis has been extensively studied in MM, there is limited information on the value of BC in SMM.

This study aimed to investigate the prognostic value of BC metrics opportunistically derived from baseline WBLDCT in subjects with SMM. We hypothesized that BC metrics assessing muscle mass and adipose tissue in both quality and quantity could serve as additional imaging biomarkers for progression.

## 2. Materials and Methods

This retrospective cohort study received approval from the Institutional Review Board (Control Number 2021P001575) with a waiver of individual informed consent and was compliant with the Health Insurance Portability and Accountability Act (HIPAA).

### 2.1. Study Cohort and Data Collection

The Picture Archiving and Communication System of a tertiary care academic hospital was searched for consecutive WBLDCT scans performed between July 2017 and March 2021. Inclusion required a diagnosis of SMM at the time of WBLDCT, a follow-up period of more than 3 months, and no diagnosis of MM within three months following the WBLDCT. Incomplete imaging and prior myeloma treatment led to exclusion. Clinical data were collected from the electronic health record. Clinical information collected within 90 days of the WBLDCT exam included patient characteristics (age, sex, body mass index [BMI]), smoking status, and a diagnosis of diabetes mellitus type II. Positive smoking status was defined as ≥5 pack-years. Laboratory parameters included serum creatine, hemoglobin, m-protein, plasma cell infiltration, and SFLCR.

The primary outcome of interest was TTP, defined as the time from when the WBLDCT was performed until disease progression to MM. Subjects without an event at the end of follow-up were censored. Outcome data were retrieved in May 2024. Diagnosis of SMM and MM was made using the IMWG criteria [10].

### 2.2. Image Analysis

Non-contrast helical WBLDCT scans were performed using helical CT scanners from multiple manufacturers, including Siemens Healthcare (Erlangen, Germany), GE Healthcare (Milwaukee, WI, USA), Canon Medical Systems Corporation (Ōtawara, Japan), and Philips Professional Healthcare (Amsterdam, The Netherlands). The acquisition parameters were as follows: section thickness of 1.5–3.0 mm, pitch between 1.0 and 1.2, rotation time of 0.4–0.5 s, field of view of 50 cm, tube voltage of 120 kVp, tube current between 16 and 199 mAs, a mean CT dose index volume of 4.5 ± 1.4 mGy, and a mean dose-length product of 628.1 ± 181.8 mGy·cm.

**Figure 1:**
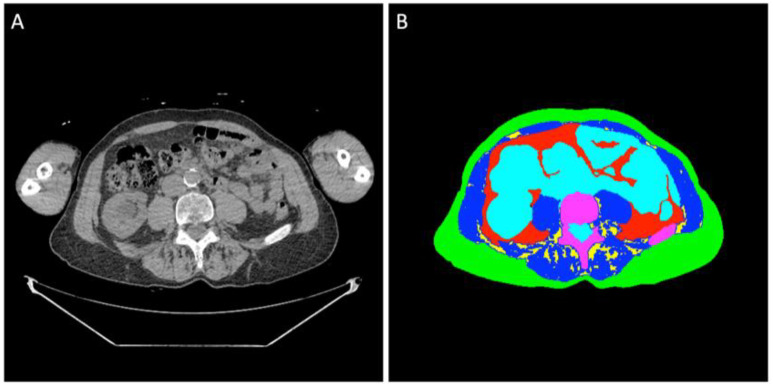
(**A**) Non-contrast WBLDCT image at L4 level of an 80-year-old man diagnosed with SMM. (**B**) Semi-automated segmentation of the abdominal tissue: green label: subcutaneous adipose tissue; red label: visceral adipose tissue; yellow label: intermuscular adipose tissue; blue label: muscle tissue; turquoise label: organs, vessels, fluids; purple label: bone.

Regions of interest of the different tissues were determined at the midpoint of the L4 vertebral body using previously validated segmentation models [8]. A standard single slice was manually determined by one of the authors (MEG) with four years of experience in medical imaging and trained by senior fellowship-trained musculoskeletal radiologists (Figure 1). All segmentations were manually inspected and corrected, if necessary, by two of the authors (MEG and IZ) using Horos DICOM viewer version 3.3.6 (www.horosproject.com (accessed on 05/15/2024)). BC analysis was conducted blinded to clinical information and outcomes.

BC analysis included the mean radiodensity in Hounsfield Units (HU) and cross-sectional area (CSA) of the abdominal subcutaneous adipose tissue (SAT), visceral adipose tissue (VAT), intermuscular adipose tissue (IMAT), and the skeletal muscle. The SAT index, VAT index, IMAT index, TAT index, and skeletal muscle index were calculated by dividing the respective CSAs by patient height in meters squared. Total adipose tissue (TAT) was calculated as the sum of the SAT, VAT, and IMAT. Skeletal muscle included all paraspinal, abdominal, paraspinal, and psoas muscle.

### 2.3. Statistical Analysis

Categorical variables were summarized as proportions. Continuous variables were summarized as medians with their respective interquartile ranges (IQR). The study cohort was divided into groups based on the sex-specific median for the respective BC metrics. Myopenia and myosteatosis were defined using the following independently established sex-specific cutoffs from the literature. For myopenia (skeletal muscle index), cutoffs were <55 cm^2^/m^2^ in men and <39 cm^2^/m^2^ in women for skeletal muscle index [29]. For myosteatosis (skeletal muscle radiodensity), cutoffs were <28.1 HU in men and <18.9 HU in women [30].

TTP with 95% confidence intervals (95%CI) was calculated using the Kaplan–Meier method. TTP of the resulting groups was compared using the log-rank test. Univariate Cox regression analysis was conducted to evaluate the association of progression risk with individual BC metrics. The results of the Cox regression analyses were presented as hazard ratios (HR) with 95% CI. BC metrics significant in univariate analysis were analyzed using multivariable regression. Multivariable analysis was performed by adding prior selected known risk factors for progression to MM as defined in the PANGEA models, adjusting for SFLCR, m-protein, serum creatinine, hemoglobulin, and age [3]. Due to data availability, the plasma cell infiltration was not included in the multivariable analysis. Groups based on the respective BC cutoff were compared for the continuous clinical parameters using the Wilcoxon rank sum test and for nominal parameters using Fisher’s exact test.

A two-sided *p*-value of <0.05 was considered statistically significant. Statistical analysis was performed using Python 3.9 with pandas, lifelines, numpy, and matplotlib packages, and using Stata 18.1 (StataCorp LLC, College Station, TX, USA).

## 3. Results

### 3.1. Baseline Characteristics

**Figure 2:**
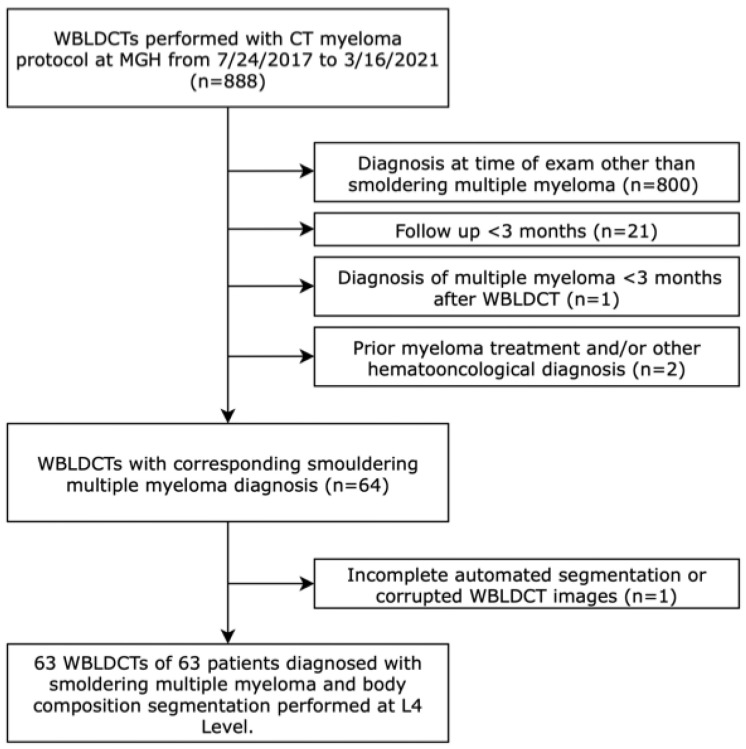
Flowchart. WBLDCT: whole-body low-dose CT.

A total of 63 WBLDCT scans from 63 subjects met eligibility criteria for BC analysis (Figure 2). The median age of the cohort was 69 years, with 41% of the study subjects being male. The median BMI was 27 kg/m^2^, and 19% of subjects had a diagnosis of diabetes mellitus. Of the entire cohort, 2% were classified as underweight (BMI < 18.5 kg/m^2^), 24% had normal weight (BMI 18.5 to <25 kg/m^2^), 46% were overweight (BMI 25 to <30 kg/m^2^), and 28% were obese (BMI ≥ 30 kg/m^2^). Further details of the baseline characteristics are provided in Table 1.

After a median follow-up of 49.2 months (95%CI: 41.6–53.7), 30 study subjects with 30 WBLDCT scans (48%) progressed to MM. The median TTP was 41.9 months (95%CI: 23.2-not reached [NR]). There was no significant difference between patients who progressed and those who did not in baseline characteristics (*p* ≥ 0.11).

### 3.2. Impact of Adipose Tissue

**Figure 3:**
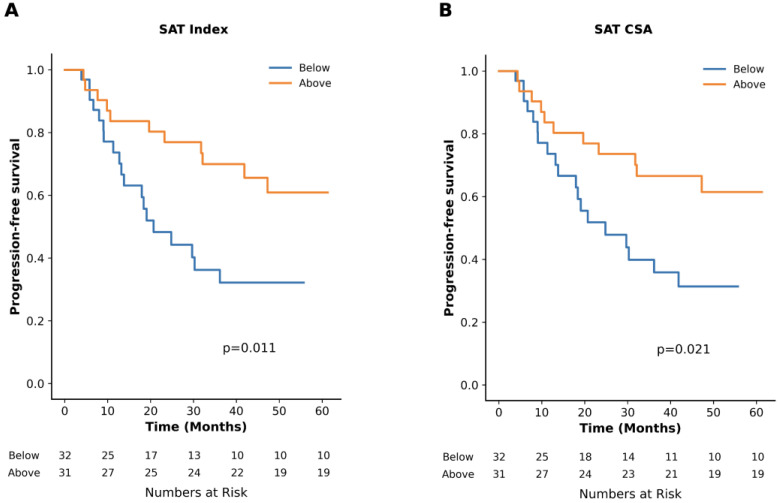
Time to progression (TTP) and subcutaneous adipose tissue cross-sectional area (SAT CSA) and index. (**A**) TTP (months) of subjects with SAT index above the sex-specific median compared to those below. (**B**) TTP (months) of subjects with SAT CSA above the sex-specific median compared to those below.

Sex-specific median values for adipose tissue BC metrics are presented in Table 2. TTP was 24.8 months vs. NR for subjects with SAT CSA below vs. above the median, respectively (*p* = 0.02). When indexed to height, TTP was 20.7 months for subjects below the median and NR for those above the median (*p* = 0.01, Figure 3). No significant differences in TTP were observed for groups divided into below vs. above the respective median on SAT radiodensity or BC metrics based on VAT, TAT, or IMAT (Table 2).

In univariate analysis, high SAT CSA (HR: 0.42 [95%CI: 0.20–0.90], *p* = 0.03) and index (HR: 0.39 [95%CI: 0.18–0.83], *p* = 0.01) were associated with a lower risk of progression to MM. In multivariable analysis, adjusting for age, serum creatinine, hemoglobin, SFLC-ratio, and m-protein, high SAT index was independently associated with a lower progression risk (HR: 0.37 [95%CI: 0.15–0.90], *p* = 0.03). There were no significant associations with progression for SAT radiodensity or BC metrics based on VAT, TAT, or IMAT above vs. below the median cutoff.

Subjects with SAT CSA above the median were younger (68.0 vs. 73.0 years, *p* = 0.05) and had higher hemoglobin levels (13.2 vs. 12.0 g/dL, *p* = 0.01). Similarly, high SAT index was associated with younger age (68.0 vs. 72.0 years, *p* = 0.03) and higher hemoglobin level (13.2 vs. 12.0 g/dL, *p* = 0.01) (Table 3). Both high SAT CSA and SAT index were associated with obesity, with 42% of subjects above the median for those BC metrics classified as obese compared to 16% below the median (*p* = 0.03).

### 3.3. Impact of Skeletal Muscle

Sex-specific median values for the BC metrics for muscle tissue are provided in Table 2. According to predefined sex-specific cutoffs for myopenia [29] and myosteatosis [30], the prevalence of myopenia was 31% in men and 11% in women, while myosteatosis was observed in 65% of men and 62% of women. Of the patients that progressed to MM, 27% had myopenia and 63% had myosteatosis, whereas 12% and 64% of the patients without progression to MM had myopenia and myosteatosis, respectively.

There was a trend for shorter TTP in patients with myopenia (18.4 months vs. NR, *p* = 0.07). TTP was 41.9 months for subjects with myosteatosis and 47.2 months for those without (*p* = 0.59). Myopenia was not associated with age, serum creatinine, hemoglobin, SFLCR, or m-protein (*p* ≥ 0.12). Among subjects with and without myopenia, 9% and 50% were classified as obese, respectively (*p* = 0.15). Myosteatosis was associated with older age (72.5 vs. 65.0 years, *p* = 0.01). No associations were found between myosteatosis and serum creatinine, hemoglobin, SFLCR, and m-protein (*p* ≥ 0.32). Additionally, 48% of subjects with myosteatosis were obese, compared to 28% of those without (*p* = 0.40).

## 4. Discussion

In this study, we demonstrated that BC metrics derived from WBLDCT using semi-automated image analysis pipelines are independently associated with TTP from SMM to MM. Our study shows that quantitatively assessed high SAT was independently associated with lower risk of progression and longer TTP and was linked to risk factors for progression.

Several studies have investigated the prognostic value of quantifying the abdominal adipose tissue in myeloma spectrum disease. Abdallah et al. reported that in 341 patients with MM, those in the lower and higher tertiles for SAT CSA had decreased overall survival compared to the middle tertile. However, TAT or VAT CSA were not associated with the outcome [19]. In a study cohort of 56 patients newly diagnosed with MM, a SAT index below the median predicted poor survival [26]. In a case-control study, Veld et al. reported that patients with MM exhibited increased TAT and SAT CSA as well as higher VAT metabolic activity in FDG-PET/CT compared to those diagnosed with MGUS [33]. In a population-based study including 300 patients with MGUS, no association between BC metrics and MGUS prevalence or progression to SMM after a median follow-up of 8 years was found [34]. Notably, discordant results in prior studies can be partially explained by variability in the study populations and different thresholds used to define BC metrics. In our study, SAT CSA and index were independently (SAT index) associated with decreased risk of progression after adjusting for risk factors for progression defined in the PANGEA models [3]. Additionally, high SAT CSA and index were associated with lower age and higher hemoglobin. These findings suggest that SAT CSA or index could serve as imaging biomarkers for assessing the risk of progression to MM in SMM. With WBLDCT routinely performed in the diagnostic algorithm for (S)MM [11,35,36], the assessment of SAT quantity could be performed opportunistically, offering potential support in clinical decision making.

VAT is more metabolically active than SAT and has a pro-inflammatory adipokine profile [37], which could potentially promote carcinogenesis [38]. For example, low levels of adiponectin, an anti-inflammatory adipokine inversely associated particularly with visceral obesity [39,40], have been linked to a higher risk of MM [41]. While increased VAT CSA was associated with adverse cytogenetics and treatment response in MM [42], our findings suggest that SAT may play a distinct, possibly protective role in SMM. It is also possible that in SMM, lower SAT could potentially be a result of increased tumor activity, where increased lipolysis and subsequent depletion of SAT could occur as the tumor progresses, ultimately leading to the diagnosis of active MM. Recent studies have highlighted that cancer-associated adipocytes in the bone marrow interact with tumor cells in a variety of metabolic mechanisms [43]. Further studies are needed to clarify the precise role of BC metrics and adipocytes and their metabolic pathways in MM progression.

The role of BMI in myeloma spectrum disease has been inconsistently reported, with some studies linking higher BMI to higher progression risk and incidence of MM [44] and MGUS [45,46,47], while others found no or limited association [31,34,48]. Additionally, in MM, being underweight [49,50] and severe obesity [50] were associated with poor survival. In our cohort, BMI and obesity were not associated with time to progression, suggesting that quantitative body composition metrics may provide a more sensitive assessment than BMI alone.

Although myopenia and myosteatosis with varying cutoffs have been associated with adverse outcomes in MM [21,22,23,25,27], results across studies are inconsistent [19,20,24,26]. In our cohort of SMM patients, neither parameter correlated with progression, suggesting that muscle alterations may become more clinically relevant in later disease stages.

Recent evidence highlights the prognostic value of advanced imaging modalities such as PET-CT and whole-body MRI in SMM, particularly for assessing focal marrow disease and early progression [32,51,52]. Importantly, body composition analysis is not limited to CT; quantitative metrics can also be derived from MRI or PET-CT, offering a complementary perspective. Incorporating BC assessment into multimodal imaging protocols may therefore enable a more comprehensive characterization of disease biology and progression risk.

This exploratory study has several limitations, including its retrospective design with potential selection bias and limited sample size. The exclusion of cytogenetic aberrations and plasma cell infiltration due to incomplete data may have influenced our multivariable analysis outcomes. Given the limited sample size, we refrained from testing alternative skeletal muscle cutoffs to avoid data-driven bias and maintain comparability with prior studies. Notably, different cutoff definitions have been shown to result in variable clinical impact on survival outcomes [53]. Additionally, our study relied on a single time point for BC analysis, which may not capture the dynamic changes in BC during disease progression. As our institution is a tertiary referral center, the exact timing of CT acquisition relative to SMM diagnosis was not available for all patients, which may have introduced bias in the time to progression analysis. Larger prospective future studies with a separate validation cohort are needed to validate our findings.

Our study demonstrated that BC analysis in WBLDCT provides prognostic information in patients with SMM. High SAT quantity was independently associated with a lower risk of progression and might serve as a predictive imaging biomarker for progression to MM in SMM. Analyzing adipose tissue composition through CT, rather than relying solely on BMI or total adipose tissue, could provide additional insights into assessing individual progression risks in SMM.

## Figures and Tables

**Table 1:** Baseline patient characteristics.

	Total	Not Progressed	Progressed	*p*-Value
WBLDCT	63	33	30	
Subjects	63	33	30	
Male sex	26 (41)	11 (33)	15 (50)	0.21
Age in years	69 (64–75)	68 (63–75)	71 (66–75)	0.42
BMI in kg/m^2^	27 (25–30)	28 (25–31)	27 (25–29)	0.44
Diabetes mellitus type II	12 (19)	9 (27)	3 (10)	0.11
Smoking ≥ 5 pack-years	24 (38)	12 (36)	12 (40)	0.80
SFLCR	13 (4–63)	9 (3–18)	27 (8–79)	0.44
Serum creatinine in mg/dL	0.9 (0.8–1.1)	0.9 (0.8–1.1)	0.9 (0.8–1.2)	0.46
Hemoglobin in g/dL	12.6 (11.7–13.6)	12.7 (11.8–13.6)	12.6 (11.7–13.6)	0.60
M-protein in g/L [n missing values]	1.3 (0.6–1.9) [13]	0.8 (0.3–1.4) [20]	1.6 (1.1–2.2) [20]	0.33
PCI in % [n missing values]	15 (10–20) [31]	15 (8–20) [5]	18 (10–20) [32]	0.26

Data is presented as median (interquartile range) for continuous variables and n (%) for categorical variables. Abbreviations: BMI: body mass index; SFLCR: serum-free light chain ratio; m-protein: monoclonal protein; PCI: plasma cell infiltration; WBLDCT: whole-body low-dose CT.

**Table 2:** Association between body composition analysis metrics and time to progression in smoldering multiple myeloma.

Body Composition Metric	Median Males (IQR)	Median Females (IQR)	TTP Based on Median in Months (95%CI): Below vs. Above
Adipose Tissue			
SAT CSA in cm^2^	213.2 (187.5, 271.1)	273.1 (185.6, 336.2)	24.8 (13.8–41.9) vs. NR (32.1-NR), *p* = 0.02
SAT Index in cm^2^/m^2^	74.4 (63.7, 93.7)	101.6 (72.9, 125.0)	20.7 (13.2–36.1) vs. NR (41.9-NR), *p* = 0.01
SAT Radiodensity in HU	−89.0 (−95.7, −79.1)	−100.5 (−104.0, −93.4)	36.1 (19.1-NR) vs. NR (18.4-NR), *p* = 0.47
VAT CSA in cm^2^	170.5 (114.4, 222.4)	128.8 (55.7, 177.0)	36.1 (19.6-NR) vs. NR (12.7-NR), *p* = 0.61
VAT Index in cm^2^/m^2^	59.3 (36.3, 76.2)	45.8 (20.4, 71.7)	36.1 (19.6-NR) vs. NR (12.7-NR), *p* = 0.61
VAT Radiodensity in HU	−96.2 (−101.6, −93.6)	−96.5 (102.0, −87.9)	36.1 (11.3-NR) vs. NR (19.6-NR), *p* = 0.38
IMAT CSA in cm^2^	3.2 (1.7, 7.1)	3.0 (1.6, 7.0)	47.2 (24.8-NR) vs. NR (12.7-NR), *p* = 0.73
IMAT Index in cm^2^/m^2^	1.3 (0.5, 2.5)	1.0 (0.7, 2.6)	47.2 (24.8-NR) vs. NR (12.7-NR), *p* = 0.76
TAT CSA in cm^2^	399.6 (337.7, 518.5)	435.9 (258.6, 494.9)	29.7 (18.4-NR) vs. NR (23.2-NR), *p* = 0.18
TAT Index in cm^2^/m^2^	133.6 (108.7, 162.2)	161.9 (101.2, 193.6)	30.3 (19.1-NR) vs. NR (13.2-NR), *p* = 0.42
Muscle Tissue			
Skeletal Muscle Index ^a^ in cm^2^/m^2^	59.8 (53.6, 66.5)	48.5 (43.6, 53.8)	18.4 (5.8–41.9) vs. NR (29.7-NR), *p* = 0.07
Skeletal Muscle Radiodensity ^b^ in HU	22.5 (18.2, 30.8)	16.2 (7.8, 20.9)	41.9 (13.2-NR) vs. 47.2 (19.6-NR), *p* = 0.59

^a^ Reference cutoff used for myopenia: skeletal muscle index < 55.0 cm^2^/m^2^ for males and <39.0 cm^2^/m^2^ for females. ^b^ Reference cutoff used for myosteatosis: skeletal muscle radiodensity < 28.1 HU in males and <18.9 HU in females. Abbreviations: CI: confidence interval; CSA: cross-sectional area; HU: Hounsfield Unit; IMAT: intermuscular adipose tissue; IQR: interquartile range; NR: not reached; SAT: subcutaneous adipose tissue; TAT: total adipose tissue; TTP: time to progression; VAT: visceral adipose tissue;

**Table 3:** Association between subcutaneous adipose tissue index and subcutaneous adipose tissue cross-sectional area and risk factors for progression.

	SAT Index			SAT CSA		
	Below	Above	*p*-Value	Below	Above	*p*-Value
Age in years	73.0	68.0	0.03	72.0	68.0	0.05
Serum creatinine in mg/dL	0.9	0.9	0.84	0.91	9.0	0.91
Hemoglobin in g/dL	12.0	13.2	<0.01	12.0	13.2	0.01
SFLCR	13.9	11.4	0.14	13.1	12.5	0.24
M-protein in g/L	1.3	1.4	0.57	13.3	14.0	0.83

Risk factors (median) are given for groups above vs. below the sex-specific median of the respective body composition metric. Abbreviations: CSA: cross-sectional area; m-protein: monoclonal protein; SAT: subcutaneous adipose tissue; SFLCR: serum-free light chain ratio.

## Data Availability

The datasets generated during and/or analyzed during the current study are available from the corresponding author upon reasonable request.

## Abbreviations

BC: body composition; BMI: body-mass index; CI: confidence interval; CSA: cross-sectional area; HU: Hounsfield Unit; IMAT: intermuscular adipose tissue; MM: multiple myeloma; m-protein: monoclonal protein; NR: not reached; SAT: subcutaneous adipose tissue; SFLCR: serum-free light chain ratio; SMM: smoldering multiple myeloma; TAT: total adipose tissue; TTP: time to progression; VAT: visceral adipose tissue; WBLDCT: whole-body low-dose CT.
